# Verification of a Rapid Analytical Method for the Qualitative Detection of *Listeria* spp. and *Listeria monocytogenes* by a Real-Time PCR Assay according to EN UNI ISO 16140-3:2021

**DOI:** 10.3390/pathogens13020141

**Published:** 2024-02-04

**Authors:** Veronica Bolzon, Michela Bulfoni, Massimo Pesando, Alessandro Nencioni, Emanuele Nencioni

**Affiliations:** 1 Biofarma Group Srl, Via Castelliere 2, 33036 Udine, Italy; bolzon.veronica@biofarmagroup.it (V.B.); pesando.massimo@biofarmagroup.it (M.P.); 2 Department of Medicine, University of Udine, 33100 Udine, Italy; michela.bulfoni@uniud.it; 3 IBSA Institut Biochimique SA, Via del Piano 29, CH-6915 Lugano, Switzerland; alessandro.nencioni@ibsa.ch

**Keywords:** listeria monocytogenes, quality control assessment, real time PCR, International Organization for Standardization (ISO), microbial contamination, food industry

## Abstract

Microbial contamination and foodborne infections are a significant global public health concern. For this reason, the detection, monitoring, and characterization of pathogens represent a significant challenge in quality control settings. Standard approaches, such as culture methods and biochemical tests, are known to be very time-consuming and intensive. Conversely, molecular technologies based on the genomic identification of bacteria are quick and low-cost. *Listeria monocytogenes* is an opportunistic pathogen and a major concern especially in food industries. It is important to understand and implement multiple quality control measures to control *Listeria* infection risk and prevent the contamination of products. Standardized detection and confirmation tests such as the API Listeria test, MALDI-TOF MS, and PCR analysis are available. The aim of our work is to provide a specific molecular method, designed according to the EN UNI ISO 16140-3:2021, for the specific detection, monitoring, and characterization of *Listeria* spp. and *Listeria monocytogenes* contamination. The verification of this new rapid approach by real-time PCR (qPCR) overcomes the limitations of culture-based techniques, meeting all the verification criteria required by ISO guidelines, including implementation and item confirmation. This system offers a powerful approach to the real-time assessment of food safety, useful for industry self-monitoring and regulatory inspection.

## 1. Introduction

Despite improvements in the production, handling, and distribution of food products, protecting consumers from bacterial pathogens, including *Listeria monocytogenes*, still remains a challenge [1,2,3,4,5].

*Listeria monocytogenes* should be identified as a hazard that can present a risk of contamination to the food industry since it can survive most common stress levels, such as high salinity, acidity, refrigeration temperatures, and low water activity [1,3,5,6,7,8]. The prevalence of *L. monocytogenes* in food production and the environment has been considered as a cause of listeriosis outbreaks. An effective control program recommended by recognized authorities must be implemented throughout all the production processes [4,6,8]. Methods for the detection and for the identification of *L. monocytogenes* should be validated by recognized national or international entities, such as the International Organization for Standardization (ISO) [9,10].

Guidelines for the detection of *L. monocytogenes* recommend stick swabs and sponges to test production areas at different points. Dry swabs should be used if samples are collected from wet surfaces, and alternatively, wet swabs with sterile diluent (e.g., phosphate-buffered saline; PBS) should be used when collecting samples from dry surfaces [11,12,13,14,15].

The development of new methods for the rapid and cheap detection of pathogens in the food industry is a priority to ensure consumer safety [16,17,18]. First of all, it is necessary to test raw materials, then the production routes, and, finally, to verify the finished product. In this way, the possibility of injury to health, and collaterally to the production quality, should be eradicated.

Although the research, enumeration, and identification of pathogenic bacterial is addressed from different aspects, recently much attention has been paid to new, more sensitive and versatile technologies to be used in quality control laboratories as a tool for monitoring production [16,19,20,21].

Traditional methods for the detection of pathogenic microorganisms can be divided into two groups. The first are quantitative methods, in which bacteria are counted and the result is expressed as the number of organisms present per unit weight of sample. The enumeration of microorganisms is generally performed using plate counts [22]. Culture methods are the most used and consist of plating dilutions of the sample on dishes containing specific nutrient media. During the incubation period, bacteria grow to form discrete colonies, which can then be counted by a specialized operator [20,21,23,24,25].

The second type of assay for microbial identification is qualitative. These methods define only the presence/absence of specific bacteria in a given sample. Qualitative procedures are used when we desire to exclude the presence of a certain microorganism in the sample. The use of enrichment procedures is often motivated by the fact that pathogenic microorganisms are present in low concentrations in food and environmental matrices and could therefore be difficult to identify using direct counting procedures. For confirmation purposes, further biochemical tests may be performed to discriminate the bacteria of interest from closely related microbial forms [19,20,21,22].

These identification methods are simple, convenient, and not wasteful. However, they have a disadvantage in that they require several days to weeks to provide a reliable result; furthermore, the phenotypic properties on which the identification of bacteria is based can be expressed ambiguously, which makes the interpretation of the results difficult. Trained and highly qualified personnel are also required to carry out these investigations. Another limitation of traditional tests is their inability to detect the presence of viable but non-cultivable cells, which is a very common physiological state [22,23,24,25,26].

To overcome these limits, new molecular technologies, such as nucleic acid amplification (PCR), have significantly improved the specificity and sensitivity of routine tests, significantly reducing the time required for the detection of microbial pathogens [27].

For the detection of microorganisms in food matrices and the environment, qualitative real-time PCR (qPCR) and semi-quantitative or quantitative or multiplex PCR (qualitative) are available [26,27,28]. Conventional PCR assays for the detection of *L. monocytogenes* in environmental and food samples generally include enrichment by culturing samples in a broth (e.g., Buffered Listeria Enrichment Broth (BLEB) or Brain Heart Infusion (BHI)) with selective agents and subculture to *Listeria* plating media. Then, the DNA is extracted from bacteria and species specification by target gene-based RT-PCR is performed [29,30].

The International Organization for Standardization (ISO)’s standard for PCR methods for the detection of foodborne pathogens (ISO 22174:2005) indicates that the presence of PCR inhibition shall be demonstrated using appropriate controls and that an internal control (IAC) or external control (EAC) should be performed in every PCR reaction [9,10].

Considering all these aspects, the intent of our work is to provide a full verification of a new rapid analytical method for the qualitative detection of *Listeria* spp. and *L. monocytogenes* by an RT-PCR assay, according to the EN UNI ISO 16140-3:2021 “Microbiology of the food chain” including both an implementation and an item verification.

## 2. Materials and Methods

### 2.1. Sample Composition

#### 2.1.1. Implementation Verification

For the implementation verification, a commercial milk powder (Neolatte 1—Unifarm S.p.a. Ravina -Italy) was selected due to its similar composition to the products’ matrices analysed in our laboratory. The specific formulation consists of a mix of powdered skimmed milk, whey, oils, maltodextrins, probiotics, proteins, salts, minerals, and vitamins.

#### 2.1.2. Item Verification

The verification of this new analytical method was performed for the detection of *Listeria* spp. and *Listeria monocytogenes* in environmental swabs. Therefore, in this step, ready-to-use environmental swabs diluted in 10 mL of Half-Fraser broth (Biocolturalab; Commercial references: Listeria ½ Fraser broth w/LP (cod. 112-151-8T) were analysed.

### 2.2. Sample Preparation

A reference quantitative strain of *Listeria monocytogenes* (ATCC 19115; 10–100 CFU/100 µL, Microbiologics—Biogenetics; Commercial references: *L. monocytogenes* (4b) (cod. 0687C) was used for both verification tests.

According to the ISO 16140-3:2021, 7 samples of 25 g milk powder were diluted with 225 mL of enrichment Half-Fraser broth (Biolife; Listeria Fraser broth Half conc. (cod. 5115943) and 3-5 CFU were inoculated under LAF hood. Another sample without *Listeria monocytogenes* was analysed as blank. In the same way, seven commercial environmental swabs diluted with 10 mL of enrichment Half-Fraser broth (Biocolturalab; Commercial references: Listeria ½ Fraser broth w/LP (cod. 112-151-8T)) were inoculated with 3–5 CFU of *Listeria monocytogens*, using another specific blank sample.

At the time of inoculation, the plate count on a non-selective medium, Tryptic Soy Agar (TSA-Biolife), of the reference strain was performed, according to ISO 7218. Two plates of two consecutive dilutions were spread for the inoculation confirmation.

After the inoculation, milk powder samples and environmental swabs were incubated for 18–20 h at 37 °C (36–38 °C), while TSA plates were incubated for 4 days at 34 °C (30–35 °C).

### 2.3. DNA Extraction

DNA was extracted from the milk powder enrichments using the SureFast PREP Bacteria (r-biopharm). For the environmental swabs, DNA extraction was performed both with SurFast PREP Bacteria and, after 26–28 h of incubation, with the Lysis buffer provided by the SureFast Listeria 3plex ONE kit, following the manufacturer’s instruction. The last extraction method was a rapid assay, based only on cell lysis.

### 2.4. Real Time PCR Amplification

The PCR master-mix was prepared according to the specific protocol described in the SureFast Listeria 3plex ONE manual. Briefly, qPCR master mix was prepared under PCR hood calculating 19.3 µL of reaction mix and 0.7 µL of Taq for each sample, and a 10% additional volume. The following conditions were applied: initial denaturation at 95 °C for 1 min, followed by 45 cycles of denaturation at 95 °C for 10 sec and annealing/extension at 60 °C for 15 s, with the maximum ramp rate.

All samples were analysed in triplicate. For the reaction, 5 controls were tested in each amplification reaction: a no-template control (NTC), an extraction control, a positive control (PC), a zero control, and a medium control.

The thermal protocol was set according to the kit’s manual amplification and analysis was performed with the QIAquant 96 5plex real-time PCR instrument (QIAGEN GmbH, Germany).

## 3. Results

### 3.1. Implementation Verification

According to ISO 7218, a spread of 10× the inoculum volume was performed to have a representative number of colonies to count. The inoculum volume was equal to 10 µL of the reconstituted reference strain. According to the declared data of the reference strain, 100 µL corresponded to 10–100 CFU of *Listeria monocytogenes*. We counted 17.5 and 23 CFU/100 µL on a non-selective medium (TSA plates).

Two plates with the inoculum volume and two plates with 10× the inoculum volume were enumerated as a confirmation of the count precision.

Plate counts were performed ensuring proportionality between the different dilutions tested. In order to calculate the average numbers of colonies for the *n* and *n* + 1 dilutions, the following equation was applied [20]:

$$N=\frac{\sum C}{V\times (1+0.1)\times d}$$

where *∑C* = sum of the average numbers obtained from the count of the colonies on the plates in the two consecutive dilutions considered; *V* = volume (mL) of the inoculated amount in each plate; and *d* = dilution factor corresponding to the first dilution considered.

Plate count results are expressed in CFU/trial portion and are given in Table 1.

Therefore, all the analysed milk powder samples can be considered valid whether they were inoculated with a number of CFU between three and five, or less than three, for the trial portion. Since the limit of acceptability (at least six out of seven positive repeats) is satisfied also with an inoculum level <3 CFU/trial portion, the results can be considered valid. In the RT-PCR reactions, all of the tested samples and controls show amplification in the VIC channel as the internal reaction control. All the experimental sample (100%) replicates showed amplification in the ROX channel, specific for the presence of *Listeria* spp. DNA.

The control medium, the zero control, the NTC, and all replicates of sample 5 and blank were negative for the presence of *Listeria* spp. All of the sample 1, sample 2, sample 3, sample 4, sample 6, and sample 7 replicates showed amplification in the Cy5 channel, specific for *Listeria monocytogenes*. The control medium, the zero control, the NTC, and all replicates of sample 5 and blank were negative for the presence of *Listeria monocytogenes*.

Summarizing, the RT-PCR experiments showed positivity for the detection of *Listeria monocytogenes* in six out of seven samples (85.7%) and negativity for the blank.

### 3.2. Item Verification

The item verification was performed on environmental swabs. Also, in this case, two plates were spread with the inoculate volume and two plates with 10× the inoculated volume were spread. Plate counts are expressed in CFU/trial portion and given in Table 1.

#### 3.2.1. Verification Using the SureFast PREP Bacteria DNA Extraction

The results obtained from an RT-PCR analysis of samples extracted using the SureFast PREP Bacteria DNA extraction were in line with the attended (Supplementary Table S1).

The qPCr results were interpreted as follows:

- VIC as the internal control of reaction.
- ROX for the detection of Listeria spp.
- Cy5 for the detection of *Listeria monocytogenes*.

According to the protocol of the kit, a sample can be considered positive for the presence of *Listeria monocytogenes* if it showed amplification in all channels or at least in Cy5 and ROX.

If there was positivity in ROX but not in Cy5, Listeria spp. were detected but there was no presence of *Listeria monocytogenes*, independently of the VIC channel.

If there was amplification in VIC but not in ROX and in Cy5, the sample was negative for the presence of *Listeria monocytogenes* and Listeria spp.

No amplification in any channels, VIC, ROX, and Cy5, corresponded to an invalid sample.

The implementation verification was considered valid if six of the seven inoculated samples were positive to the presence of Listeria spp. and *Listeria monocytogenes* and if the blank was negative.

If the blank was positive, all the analyses were repeated.

Furthermore, if the inoculum level was >5 CFU/trial portion, the results could not be considered, and the experiment was repeated.

If the inoculum level was <3 CFU/trial portion and the limit of acceptability (at least six out of seven positive repeats) was satisfied, the results were considered valid.

All samples and controls (100%) showed amplification in the control VIC channel as the internal reaction control. Considering the ROX channel, referred to as the *Listeria* spp. Target, all the samples and respective replicates (100%) were amplified with valid Ct values. Also, the positive control and the extraction control were positive for the presence of *Listeria* spp.

The medium control, the zero control, the NTC, and all the replicates of the blank were correctly negative. Regarding the specific detection of *Listeria monocytogenes* DNA, all of the samples (100%) were identified as positive, confirming the analytical power of the screening test.

#### 3.2.2. Verification Using Rapid DNA Extraction

For all the verifications conducted with RT-PCR starting from DNA extracted with the rapid method, a valid result was obtained. The observed data were all in line with the expected ones. All DNA showed amplification in the internal control channel (VIC) and in the *Listeria* spp. and *Listeria monocytogenes*-specific amplification reaction.

In total, 100% of the samples tested by RT-PCR were positive for the detection of *Listeria monocytogenes* with both the extraction methods.

Therefore, all the analysed samples can be considered valid for a quality control intent, whether they were inoculated with a number of CFU between three and five, or less than three, for the trial portion, satisfying the limit of acceptability.

## 4. Discussion

In this study, we performed a strength verification of an RT-PCR method for the detection of pathogenic *Listeria* spp. and *L. monocytogenes* in environmental samples. The method focused both on an implementation and on an item verification, according to the EN UNI ISO 16140-3:2021 “Microbiology of the food chain—Method validation—Part 3: Protocol for the verification of reference methods and validated alternative methods in a single laboratory” [9,10]. In this way, we demonstrated that our laboratory could run the supplier-validated method correctly and that the user laboratory can run the method with the items claimed. In the future, with other item verifications, this method can be employed to monitor, in a fast and cheap way, all of the production line, from raw materials to finished products. Our verification has demonstrated that an already validated method performs, in another user’s hands, according to the method’s specifications and is fit for its intended purpose.

*Listeria* spp. are widely found in food and in the environment and *L. monocytogenes* could represent a cause of listeriosis in humans [1,2,3,4,5]. Therefore, the development of a PCR-based method for the species-specific detection of its pathogenic DNA provides an independent screening tool to monitor the quality and the safety of the production [10,11,13].

The RT-PCR results obtained were in line with the manufacturer’s validation data and with ISO guidelines [9,10]. Expected positivity results for the targeted *Listeria* genomes were detected. In particular, since 85.6% of the spiked samples were positive for the presence of *Listeria* spp. and *L. monocytogenes*, the implementation verification was considered as valid [9]. In the case of item verification, 100% of the inoculated samples were positive.

Considering the procedure workflow, the DNA extractions both with the SureFast PREP Bacteria and with the rapid extraction showed the same performance. So, for routine analysis of environmental swabs, it is possible to apply the rapid method to further shorten the time to result.

Even though a plethora of qPCR-based methods for the detection of *L.* monocytogenes have been reported, most of them lack proper validation, which is necessary to encourage their use by the food industry as monitoring tools. Covering this gap, the aim of our qPCR assay was to save time by reducing the turnaround time of the analytical report by verifying the performance of a molecular kit according to ISO guidelines.

In other scientific works, PCR or qPCR methods were developed for the detection and quantification of *L.* monocytogenes from different food matrices, but with some limiting aspects. For example, Rantsiou et al. [31] designed a qPCR for meat products and from cured ham, while Rodríguez-Lázaro et al. [32] reported a quantification assay of pathogens from pork and salmon samples. In these works [31,32], protocols started from the product matrix, affecting the sensitivity, LOD, and LOQ of the assay. In order to overcome this limitation, we added a first pre-enrichment step in culture to amplify the starting concentration of microorganisms, if present. Also, Hough et al. [33] developed a qPCR method for *L. monocytogenes* in cabbage, with a primary centrifugation step for the concentration which extended the range for the pathogen quantification. Unfortunately, the sample concentration could cause an increase in PCR inhibitors, affecting the amplification PCR reaction. Pre-PCR steps by filtration have been proposed [34] for the isolation of bacterial DNA in yogurt or by a lysis procedure in dairy products and eggs.

Taking these aspects into account, in our work we included pre-enrichment in culture, as recommended by the supplier, to increase the sensitivity of the assay and to carry out only one lysis of the product matrix, without concentrating it to resurrect the inhibitors. All this was verified using milk powder, which simulated the complexity of the matrices being analysed.

This proposed *Listeria*-specific approach met the specifications of existing molecular detection methods for the identification of pathogens in food and the production environment and also satisfies ISO rules [9,10]. Using powder milk, we simulated the complexity of the matrices being analysed, putting us in a difficult position.

The verification performance of the *Listeria* spp. and *L. monocytogenes* test satisfied all of the assessment criteria, with good results for the declared validated parameters [9,10].

The interpretation rules for single (*Listeria monocytogenes*)- or double-positive (*Listeria monocytogenes* and *Listeria* spp.) target genes will lead to differences in the interpretation of positive results (negative/positive).

Despite the fact that a full internal validation report of the SureFast Listeria 3plex ONE kit is already provided by the supplier, validation certifications by international organizations are expected to be developed in 2024.

Furthermore, these observations indicate that the RT-PCR assay could be used to detect pathogenic *L. monocytogenes* in samples more rapidly than using the standard culture method (4–7 days) in a standardized manner [23,25,26,27,28,29]. In summary, verification of the RT-PCR test was consistent with the product requirements, and the detection system meets the detection performance stated in the kit. Finally, we hypothesize that this study will be useful for other laboratories before using a new analytical system for the molecular detection of pathogenic microorganisms.

## 5. Conclusions

In conclusion, we have verified an RT-PCR analysis to identify *Listeria* spp. and *Listeria monocytogenes* DNA in environmental samples. The results of the assay are consistent with the validation data of the manufacturer of the commercial kit.

This assay can be applied for the rapid screening and detection of pathogenic bacteria in food processing environments, providing accurate results to improve the microbiological safety of production.

## Figures and Tables

**Table 1:** Results obtained from plate count method of milk powder samples and environmental swab inoculums.

	Inoculation (CFU)		10× Inoculation (CFU)		Result (CFU/Trial Portion)
	plate 1	plate 2	plate 1	plate 2	
milk powder sample 1	4	3	23	24	2.5
milk powder sample 2	2	3	30	32	3.0
milk powder sample 3	2	1	16	18	1.7
milk powder sample 4	4	1	17	13	1.6
milk powder sample 5	1	0	16	17	1.5
milk powder sample 6	3	2	18	15	1.7
milk powder sample 7	0	2	26	24	2.4
environmental swab 1	3	3	20	20	2.1
environmental swab 2	1	2	17	21	1.9
environmental swab 3	1	2	19	22	2.0
environmental swab 4	2	1	20	12	1.6
environmental swab 5	3	2	26	20	2.3
environmental swab 6	3	2	15	21	1.9
environmental swab 7	1	0	17	16	1.5

## Data Availability

The authors confirm that all relevant data are included in the article and materials are available on request.

## Supplementary Materials

The following supporting information can be downloaded at: https://www.mdpi.com/article/10.3390/pathogens13020141/s1, Supplementary Table S1: Raw Ct values obtained by qPCR.
